# Does egg carotenoid improve larval quality in Arctic charr (*Salvelinus alpinus*)?

**DOI:** 10.1002/ece3.8812

**Published:** 2022-04-11

**Authors:** Torvald Blikra Egeland, Einar Skarstad Egeland, Jarle Tryti Nordeide

**Affiliations:** ^1^ Faculty of Biosciences and Aquaculture Nord University Bodø Norway; ^2^ Faculty of Education and Arts Nord University Bodø Norway

**Keywords:** Arctic charr, carotenoid, female ornaments, ornaments, *Salvelinus alpinus*, signal

## Abstract

Females in mutually ornamented species are often less conspicuously ornamented than their male conspecifics. It has been hypothesized that offspring quality may decrease if females invest more resources into ornaments at the expense of resources in eggs. An experiment was carried out to test whether natural variation in carotenoid in the eggs from a wild population of Arctic charr (*Salvelinus alpinus*) was associated with survival and growth of their offspring until hatching. Wild Arctic charr were caught at a spawning ground during the spawning period. Eggs from two different females, one female with yellowish carotenoid‐rich eggs and one with paler eggs, were fertilized by sperm from the same male. This was repeated until gametes were collected from 42 females and 21 males, giving a total of 21 groups. After fertilization, the zygotes from each of the two females were reared in four replicated groups. These 168 groups were reared separately until hatching when the surviving larvae were counted and their body length measured. For the two response variables survival and body length at hatching, no effect was demonstrated of any of the predictors (i) amount of carotenoid in the unfertilized eggs, (ii) the mothers' body condition, or (iii) ornament intensity of their red carotenoid‐based abdominal ornament. Thus, this study gives no support for the hypothesis that females investing less carotenoid into their eggs suffer from decreased offspring quality until hatching. This lack of association between female ornament intensity and their fitness is not as expected if female ornaments evolved due to direct sexual selection from males on the more ornamented females (“direct selection hypothesis”).

## Graphical abstract

We test whether females Arctic charr (*Salvelinus alpinus*) investing less carotenoids in their eggs suffer from decreased zygote ‐ larval survival and growth until hatching. Wild charr were caught at a spawning ground during the spawning season. The charr were stripped for gametes. In each trial we fertilized eggs from one female with relatively strong yellowish carotenoids‐rich eggs and from one female one with relatively pale eggs. Eggs from each of the two females were fertilized by sperm from one randomly chosen male. This was repeated until four groups of eggs (replicates) from each of the two females were fertilized. This four times replicated paternal half‐sib experimental design was carried out with 21 trials including 42 females and 21 males producing 168 groups of zygotes. The 168 groups were reared separately until hatching, when survival and length of the offspring was estimated. Statistical analyses revealed no effect on neither of the two response variables survival and growth at hatching (analyses in two separate model‐runs), on none of the predictors (i) amount carotenoids in the unfertilized eggs, (ii) the mother's body condition, or (iii) intensity of their red carotenoid‐based abdominal ornament. Thus, this study gives no support for the hypothesis that females investing carotenoids into their ornamented belly suffer from decreased offspring quality.

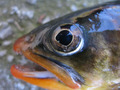

## 1 INTRODUCTION

Females of species with conventional sex roles are often ornamented although usually less elaborately compared to their male conspecifics (Darwin, 1871). Several hypotheses have been proposed to explain this difference between the sexes. Females spend more time and energy on gamete production, gestation, and parental care. This leads to an operational sex ratio skewed towards males, females choosing among different mates more than males do, and higher variation in reproduction success between males than between females. Males more than females will have to signal their quality through conspicuous signals to be chosen as a mate (Clutton‐Brock & Vincent, 1991; Emlen & Oring, 1977). Conspicuously ornamented females may have higher survival cost in some species where the sexes differ in parental care (Darwin, 1871; Heinsohn et al., 2005). An alternative hypothesis assumes condition‐dependent ornaments and suggests that females allocating resources into ornaments do this at the cost of resources invested in eggs or offspring (Fitzpatrick et al., 1995; Lande, 1980). Males choosing to fertilize eggs from such ornamented females do so at a fecundity cost which will constrain further exaggeration of female ornament evolution. However, it may still benefit males to choose such ornamented females if (i) the cost of ornaments is lower for high compared to low quality females, or (ii) the sexually attractiveness of the offspring of the ornamented mother more than outweighs her fecundity cost (Grafen, 1990; LeBas, 2006; Simmons & Emlen, 2008; Watson & Simmons, 2010; Zahavi, 1975).

Female ornaments—either female‐specific or mutual ornaments—may have evolved as a result of direct selection from males on more ornamented females (reviewed by Amundsen, 2000). This “direct selection hypothesis” predicts males to prefer ornamented females, as well as a positive relationship between female ornaments and fitness. The “genetic correlation hypothesis” suggests that female ornaments in mutually ornamented species are due to a non‐adaptive genetic correlation arising as a consequence of sexual selection on males and sharing of most genes by both sexes (Lande, 1980). Predictions are that males should court or mate with drab females or not have a preference at all, and a negative or no association between female ornaments and offspring quality. Finally, female ornaments signal female dominance and evolved through female‐female competition over resources according to the “social selection hypothesis” (Heinsohn et al., 2005, LeBas, 2006; reviewed by Tobias et al., 2012). Fourth and fifth, female ornaments may evolve simply to advertise readiness to reproduce, or as warning signals (aposematism).

Conclusions in empirical studies on evolution of ornaments in females of mutually ornamented species are ambiguous, as reviewed by Amundsen (2000), Kraaijeveld et al. (2007), Clutton‐Brock (2009), Nordeide et al. (2013), and Svensson and Wong (2011). This ambiguity has been suggested to be due to between‐species variation in ornament‐fecundity relationships deciding whether or not male mate choice is adaptive (Watson & Simmons, 2010). Studies published in the later years give ambiguous conclusions as well, with some studies being in accordance with (Cantarero et al., 2017; Cotton et al., 2015; Hernández et al., 2021; Lüdtke & Foerster, 2018, 2019), and others contrary to (Caro et al., 2021, and Rigaill & Garcia, 2021) predictions from the direct selection hypothesis. Some studies give support to the genetic correlation hypothesis (Sganga & Greco, 2019), and the social selection hypothesis (Enbody et al., 2018, see also Kroken et al., 2021). A few studies simply suggest female ornaments to signal readiness to reproduce (e.g., Belliure et al., 2018; Laplante, 2015).

Carotenoid is present in both ornaments and eggs in several fish species. The genetic correlation hypothesis has received some support from research groups studying female ornaments and their egg quality in fishes. Of the four studies which had quantified female carotenoid‐based ornamentation and carotenoid content of their eggs as reviewed by Nordeide et al. (2013), two studies reported a negative association (Nordeide et al., 2006; Ramstad et al., 2010), two reported no significant association (Garner et al., 2010; Nordeide et al., 2008), whereas no study reported a positive association. In the last few years, one more study reported negative association between the same two parameters in brown trout (*Salmo trutta*) (Wilkins, Marques da Cunha, et al., 2017). Moreover, Janhunen et al. (2011) found negative association between the expression of carotenoid‐based ornamentation and offspring survival in female Arctic charr (*Salvelinus alpinus*). In addition, male sticklebacks (*Gasterosteus aculeatus*) seem to court reddish females relatively less (Nordeide, 2002), or show no preference at all of such carotenoid‐based ornaments (Wright et al., 2015). Neither do reddish throat or reddish pelvic spines in female sticklebacks seem to signal female‐female aggression (Yong et al., 2018) or competitive advantage (Yong et al., 2015). Finally, the genetic correlation hypothesis assumes a common genetic architecture since female ornamentation evolves by a non‐adaptive genetic correlation as a consequence of sexual selection on males. A quantitative trait analysis indeed revealed such a common architecture for genes coding for the reddish throat and pelvic spines in three‐spine sticklebacks (Yong et al., 2016). All in all, these studies give some support for the genetic correlation hypothesis to explain the evolution of female ornaments in some fish species.

Carotenoid plays important roles in several branches of biology, like physiology, immunology, biochemistry, and behavioral ecology. Carotenoid is considered beneficial and may increase immune function and antioxidant capacity, signaling aggression and dominance, as well as sexually selected conspicuous ornaments (Andersson, 1994; Blount et al., 2000). Animals cannot synthesize carotenoid themselves and must rely on carotenoid from their diet. This, but also the multiple roles of carotenoid, have led scientists to assume that access to carotenoid is limited (discussed by Monaghan et al., 2009; Svensson & Wong, 2011), at least in some habitats in the wild. Prior to the reproductive season, salmonids transport carotenoid from the flesh to eggs and skin (Crozier, 1970; Hatlen et al., 1996; Steven, 1949). This transport to the eggs and the high carotenoid content in the eggs, both suggest a beneficial role of maternally derived carotenoid also on egg and larva development. This may be especially beneficial in species like salmonids with large eggs and long development times (Ahmadi et al., 2006; Janhunen et al., 2011; Tyndale et al., 2008). The intense lipid metabolism in a O_2_ rich environment may rely on exogenous antioxidants such as carotenoid, especially before the embryo has had time to synthesize endogenous antioxidants themselves. Thus, a strong selection pressure is expected on females to invest carotenoid in their eggs instead of wasting such valuable resources on ornaments. Several studies have confirmed advantageous effects of carotenoid on egg and larva quality (e.g., Ahmadi et al., 2006; Bazyar Lakeh et al., 2010; Blount et al., 2000; Boonyaratpalin & Unprasert, 1989; Christiansen et al., 1995; Craik, 1985; Lehnert et al., 2016, 2018; Parolini et al., 2018; Rajasingh et al., 2007; Salze et al., 2005; Tyndale et al., 2008; Vassallo‐Agius et al., 2001; Verakunpiriya et al., 1997; Watanabe & Miki, 1993; Wilkins, Marques da Cunha, et al., 2017) and several others. Yet, no consensus exists, since several other authors have reported no or little effect of egg carotenoid on offspring quality (e.g., Ahmadi et al., 2006; Christiansen & Torrissen, 1997; Craik & Harvey, 1986; Kolluru et al., 2006; Svensson et al., 2006; Torrissen, 1984; Tveranger, 1986). Even if carotenoid does have a beneficial role, this effect seems non‐linear with regard to the carotenoid concentration. Craik (1985) suggested a critical level for improved effect on survival above 1–3 μg carotenoid g^−1^ eggs. Contrary, Tyndale et al. (2008) suggested no beneficial effect of carotenoid on survival below but not above 2 μg g^−1^ eggs. Too high carotenoid concentration in eggs may be disadvantageous due to its toxic effects according to Wu et al. (2017) and Amengual et al. (2011), and the conspicuously colored (yellow, orange, or red) eggs might suffer from increased predation as well (Lehnert et al., 2017).

Arctic charr is a salmonid where both sexes often are mutually and beautifully ornamented by orange to reddish carotenoid‐based color at their ventral and lower lateral parts, males more elaborately than females. Breeding specimens gather annually at specific spawning grounds (Brattli et al., 2018; Figenschou et al., 2004; Sigurjonsdottir & Gunnarsson, 1989). A few large males adopt a dominant strategy trying to defend females entering the spawning ground from several smaller, subordinate males by aggressively chasing them away. Several dominant males seem to patrol different parts of the same spawning ground simultaneously, and it is unclear to which degree the arriving females actively chose among the males (Brattli et al., 2018; Sørum et al., 2011). A negative association between female Arctic charr coloration and her offspring survivorship was reported by Janhunen, Kekäläinen, et al. (2011), using specimens kept in a hatchery and fed *ad libitum* with carotenoid‐rich (astaxanthin content 80 mg kg^−1^) salmonid food. In contrast, wild freshwater Arctic charr from oligotrophic lakes likely have lower access to carotenoid in their diet and may not get sufficient amounts. Females from a wild population of Arctic charr from Lake Soløyvatnet in North Norway, exhibit a large variation in the degree of orange‐reddish color of their skin and yellowish‐orange eggs (Nordeide et al., 2008). A negative trend, marginally non‐significant, has previously been demonstrated between the carotenoid‐based coloration and the carotenoid‐content of their eggs of females from this population (Nordeide et al., 2008). We designed an experiment to test whether the amount of carotenoid in the eggs was associated with quality of the eggs from this population. To test this, we captured wild Arctic charr at a spawning ground during the spawning period, and fertilized eggs from pairs of females—one with more yellowish eggs and another with paler eggs—with sperm from the same male and reared the zygotes until hatching. The carotenoid‐based ornament and the amount of carotenoid in the eggs of the specimens represent natural variation (not manipulated), contrary to the study by Janhunen, Kekäläinen, et al. (2011). We predicted higher quality of carotenoid‐rich eggs relative to eggs containing less carotenoid, measured as survival and body length of larvae at hatching.

## 2 MATERIALS AND METHODS

### 2.1 Fish sampling and handling

Arctic charr were caught at three different evenings during October 5–12, 2015, which is during the spawning period, from a landlocked population in Lake Soløyvatnet, an oligotrophic lake situated at 67°17′N, 14°25′E in Bodø, Northern Norway. The charr were caught by gill nets at a spawning ground and the nets fished for 2–3 h before they were retrieved 10 p.m. The fish were stored alive in tanks with frequently replaced lake water until further handling within the next 2 h. Handling of each fish started with anesthetizing each fish with MS222 and then sacrificing it with a blow to its head. Each fish was placed at a standard position in a stand and their ventral part was photographed, together with a reddish cardboard for calibration purposes using an Olympus OM‐D E‐M10, M. Zuiko Digital ED 60 mm 1:2.8 lens and a Nissin i40 flashlight. The camera and flashlight were mounted on a tripod to ensure standard distances to the fish. These photos were later used to quantify the intensity‐of‐red at the fish's abdomen (*I*_R_‐abdomen) (see “Quantifying color”). Males were stripped for semen which were stored in Eppendorf tubes. Females were stripped for eggs and ovarian fluid which were photographed in a Petri dish by the same camera and flashlight (see above) mounted on a tripod. These photos were later used to quantify the intensity‐of‐red of the eggs (*I*_R_‐eggs) (see “Quantifying red and yellow color”). The eggs were then stored in plastic containers. The eggs and sperm were transported to the hatchery where the eggs from each female were divided into two equal batches. One batch was poured into borosilicate glasses and stored at −40°C under nitrogen, to prevent carotenoid degradation, for later HPLC analysis (see Carotenoid analysis). The second batch of eggs was used for fertilizations (see Fertilization and hatchery).

Total length of the specimens was measured to the nearest 0.1 cm (24.2 cm ± 2.5, mean ± SD), and mass to the nearest 0.1 g (151.4 g ± 43.4). The weight–length relationship of the specimens was estimated by linear regression from the equation: ln*W* = ln a + b ln*L*, where *W* = total body mass (g) after stripping for gametes, and *L* is the total length (cm) as recommended by Le Cren (1951) and Froese (2006). Estimated values of the constants *a* and *b* are 0.0286 and 2.688, respectively. The relative condition factor (*K*_rel_) is *K*_rel_ = W/(0.0286 * *L* ^2,688^) was used when comparing weight–length relationship between individual specimens (Froese, 2006); Le Cren, 1951.

### 2.2 Quantifying red and yellow color

Three different methods were used to quantify red or yellowish coloration of the females and eggs. First, we lined up next to each other the glass‐beakers containing eggs from the females, and ranked them stronger yellowish to pale based on our (TBE and JTN) perceptions. This method is hereafter termed “color‐by‐vision.” Second, “Intensity‐of‐red” (*I_R_*) was obtained from digital photos of (i) the eggs (*I_R_*‐eggs) (see “Fertilizations and hatchery”) and (ii) the specimens' abdomen (*I_R_*‐abdomen) (see “Fish sampling and handling” and Appendices S1 and S2). Each of ten eggs from each female were picked at random from the photos and analyzed by Adobe Photoshop Elements 13. For each egg, the value for red, blue, and green were noted from the pixels enclosed by drawing a circle inside and close to the edge of the egg, after removing reflexes from the flashlight. The *I_R_*‐value was estimated (see below) and the mean *I_R_*‐eggs was calculated for each female. Moreover, a standardized part of the abdomen of each specimens was enclosed for each specimen according to Skarstein and Folstad (1996), and *I_R_*‐abdomen was calculated for each fish (see below). The same procedure was used to quantify red, green and blue from a standardized cardboard in order to adjust for potential between‐photos‐variation in exposure in both eggs and abdomen. The Intensity‐of‐red (*I_R_*) was calculated as *I_R_* = red/(red + green + blue) (see Neff et al., 2008; Nordeide et al., 2006, 2008; Skarstein & Folstad, 1996; Villafuerte & Negro, 1998; Yong et al., 2013). Third, mass of different carotenoids was quantified by HPLC (see “Carotenoid analysis”) and hereafter termed “carotenoid‐by‐HPLC”. We used all three methods to quantify color (or carotenoid) of the eggs to check the correlations between them (see results and Appendices).”Color‐by‐vision” was only used to select the eggs from pairs of females for fertilization and is not a quantitative measure. Color‐by‐vision is therefore not included as a predictor in the statistical models.

### 2.3 Fertilizations and hatchery

Artificial fertilization was carried out at the lab at Nord University within 4 h after stripping of the gametes. The eggs from the different females were ranked relatively to each other based on the intensity of their yellowish color when the eggs were still in the glass‐beakers (not from the photos in Appendix S1, see “color‐by‐vision” in “Quantifying red and yellow color”), and relatively stronger “yellowish” eggs from one female were paired with “pale” (appearing less yellowish) eggs from another female. Retrospect analyses showed a significant difference in the intensity‐of‐red color of the eggs (*I_R_*‐eggs) between the two categories of stronger yellowish and paler eggs (paired *t*‐test, *t*_20_ = −5.11, *p* < .0001, Appendix S3). Measured as “carotenoid‐by‐HPLC” (see Carotenoid analysis), the mean of the pale batch of eggs (“color‐by‐vision”) in each pair of eggs was relatively low compared to the mean of the stronger yellowish batch of eggs in each pair. The difference between the groups was slightly non‐significant (paired *t*‐test, *t*_20_ = −1.99, *p* = .06, Appendix S4).

The batch of eggs aimed for the fertilization experiment (as opposed to the batch aimed for HPLC analysis) from each of the two paired females was divided into four replicated groups. This resulted in four replicated groups of stronger yellowish eggs and four of paler eggs from each pair of females. The eggs surrounded by the ovarian fluid in each of these eight groups were fertilized under water by sperm from one randomly chosen male. Thus, the gametes of two females and one male gave four replicates of yellowish zygotes and four replicates of paler zygotes, and all eight batches were fertilized by sperm from one particular male in this four replicates split‐brood design. This procedure was repeated until the gametes from 42 females and 21 males were fertilized, giving a paternal half‐sib designed experiment with a total of 168 fertilizations and hence 168 groups of zygotes. Each of the 168 groups of zygotes was transferred to a separate plastic cup and reared separately. The location of each cup relative to the other cups was randomized. The cups had a bottom made of nylon mesh allowing water to flow between the eggs and out through the bottom of the cup (see details in Egeland et al., 2015). The mean (±SD) number of eggs in each cup was 51.7 (±23.98). Each cup was monitored visually every day and hatched alevins were removed, anesthetized and sacrificed by an overdose MS222, and photographed with a measuring tape for calibration purposes, by a Canon PowerShot G9 digital camera with a Canon 580EX II flash in a dark box. Length of the larvae were measured from photos. Mean hatching success was 54.6% (2,931 of 5,369 eggs). Unfertilized, infected and dead eggs, dead eyelings, and dead alevins were counted and removed once a week to prevent fungus growth (see Appendix S5).

### 2.4 Carotenoid analysis

**FIGURE 1:**
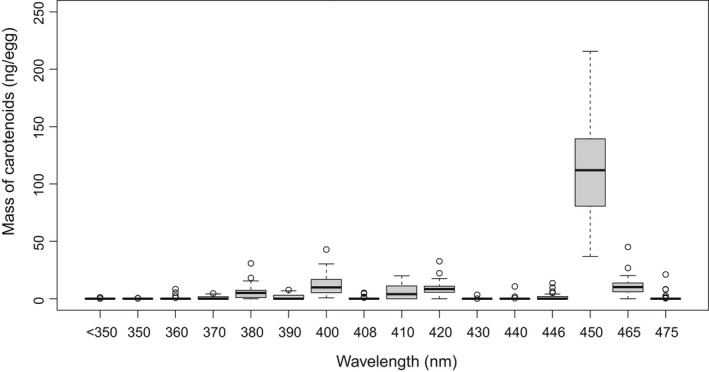
Box‐whiskers plot showing mass of carotenoid at different wavelengths (nm) quantified by HPLC. The numbers are averaged per egg and per female for all 42 females. Carotenoid at wavelengths around 440, 450, 465, and 475 nm are tunaxanthin, zeaxantin, adonixantin/idoxantin, and astaxanthin/adonirubin/cantaxanthin, respectively. Carotenoids with wavelengths <440 nm are degraded carotenoid, whereas those >440 nm are non‐degraded carotenoid. Carotenoids >400 nm look yellowish to red to the human eye

The carotenoid‐by‐HPLC was analyzed by high‐pressure liquid chromatography (HPLC) using a similar approach as in Hooker et al. (2012, chapter 4). The carotenoid in the eggs of each female were extracted using acetone in a three‐step procedure (3 × 50 ml) for a minimum of 72 h in a refrigerator (+2°C) under nitrogen, followed by evaporation of the extracts to dryness to prevent carotenoid degradation, and redissolved. Before HPLC analysis the extract was filtered and pipetted into a 2 ml HPLC vial, flushed with nitrogen and capped. The HPLC analyses were performed on an Agilent 1200 HPLC instrument with diode array detector, thermostat autosampler with enlarged injection loop, vacuum degasser, thermostat column compartment, and quaternary pump. Two identical C_18_ columns were used (ACE 5 C_18_ part no. ACE‐121‐2546) and kept at a stable temperature of 25°C. A gradient system as followed, continuously added 0.1% hexane; 0 min: 1:4 1 M ammonium acetate: methanol; 60 min: 7: 3 methanol:acetone; 100 min: 3:5:2 methanol:acetone:hexane; 110 min: 2:3 acetone:hexane; 120 min: methanol; 130 min 1:4 1 M ammonium acetate: methanol, was used as eluant. The injection volume was set at 50 μl with detection wavelength at 380, 420, 450, and 480 nm. For the calibration, standards (lutein, zeaxanthin, antheraxanthin, adonixanthin, idoxanthin, astaxanthin, adonirubin, and canthaxanthin) were each dissolved in a pure solvent of known volume. The spectrum was recorded using a common spectrophotometer, and the standard solution was injected into the HPLC instrument in a 1:1, 1:5, 1:25, and 1:125, mixture with the appropriate solvent (Mercadante et al., 2004), the injection volume was 50 µl for all mixtures. A linear calibration line was made using the HPLC software. The egg samples were analyzed in random order and spectra for each pigment peak were checked manually, to ensure that no incorrect identities were reported. Both spectral shape and wavelength were checked to detect any impurities and the peak borderlines and baseline were checked to ensure a precise calculation of the area of each peak. We decided to use the total amount of carotenoid (carotenoid‐by‐HPLC) as a predictor, with ng/egg as unit of measure, in our models (Figure 1).

### 2.5 Statistics

All statistical analyses were performed using R version 3.6.1 (R Core Team, 2019). Linear regression models were used to test for significant relationships between total amount of carotenoid‐by‐HPLC analysis of the eggs on one hand, and each of the parameters intensity‐of‐red of the eggs (*I*_R_‐eggs), intensity of red of the abdomen (*I*_R_‐abdomen) and condition‐factor (*K_rel_*) on the other hand. We fitted a mixed‐effect model using the glmer function in the lme4 package (Bates et al., 2014), to test if the response variable “hatching success” was affected by different predictors. The response variable hatching success (i.e., proportion of survivors from each of the four replicates) was entered as a proportion with the cbind function (see Crawley, 2012, p. 628 for further details) and as a consequence we ran the model with a binomial distribution. The predictors were (i) amount of carotenoid in the eggs (carotenoid‐by‐HPLC, ng carotenoid/egg, see “Quantifying red and yellow color”), (ii) intensity‐of‐red of the eggs (*I*_R_‐eggs), (iii) intensity‐of‐red of the abdomen (*I*_R_‐abdomen), and (iv) condition factor (*K*_rel_). Female‐ID and male‐ID were entered as random factors (female‐ID within male‐id). By using the function dredge in the MuMIn package (Barton, 2022) with a complete model set (including all the above‐mentioned predictors and their interactions) we compared and ranked all possible models based on the corrected Akaike Information Criterion (AICc) (Burnham & Anderson, 2002). In other words, the model selection aimed to compare the quality of the models with the trade‐off between parsimony and goodness‐of‐fit criteria based on an information theoretic approach (Fisher et al., 2018). Only models with delta ≤2, which show “substantial evidence for the models” (Burnham & Anderson, 2002), are presented (see Appendices S6 and S7 for models with delta >2). The lmer function in lme4 package (Bates et al., 2014) was used to test if the response variable “length of the larvae at hatching” was affected by the same combination of predictors and with the same approach for model selection described above.

Prior to model selection, the data were explored following Zuur et al. (2010) to check for normality, collinearity, heteroscedasticity, and outliers in the data. To avoid problems with collinearity the variance inflation factor (VIF) was calculated in models that included both total amount of carotenoid‐by‐HPLC and *I*_R_‐eggs. None of the models had VIF above 2 and model 6 (see Table 1) had VIF = 1.4 (Zuur et al., 2010). The abdomen of one of the 42 females (see above) in a pair of females was, by a mistake, not photographed. Thus, both females in this trial were excluded from the analyses where the parameter «Intensity of abdomen *I_R_* » was included (*N* = 40 for this parameter). The number of females with larvae alive at the time of termination of the experiment was 30 (12 females left no surviving offspring at the time of hatching). Thus, *N* = 30 for the parameter “length of the larvae at hatching.” All figures were produced with the ggplot2 package (version 3.3.4, Wickham, 2016).

**TABLE 1:** Test statistics from generalized linear mixed models with egg hatching success as the response variable

Model	Intercept		*K*_rel_		*I*_R_‐eggs		Carotenoid‐by‐HPLC		df	logLik	AICc	Delta	Weight
	*β*	*p*	*β*	*p*	*β*	*p*	*β*	*p*					
1	−2.15	.0017							3	−453.873	913.9	0.00	0.186
2	−16.53	.110			29.63	.164			4	−452.935	914.1	0.23	0.167
3	−4.68	.0244					0.01	.1945	4	−453.069	914.4	0.50	0.146
4	3.44	.587	−5.60	.376					4	−453.501	915.3	1.36	0.095
5	−10.95	.397	−4.60	.472	27.60	.203			5	−452.690	915.8	1.87	0.073
6	−13.62	.220			20.52	.413	0.01	.516	5	−452.744	915.9	1.98	0.070

Note

The six different models shown differ in the combinations of the predictors (i) condition factor (*K*_rel_), (ii) intensity‐of‐red eggs (*I*_R_‐eggs), and (iii) the amount of carotenoid in eggs (carotenoid‐by‐HPLC). The table shows the models with delta Akaike's information criteria (AICc) ≤2. *β* represents the estimate and *p* the *p*‐value. *I*_R_‐abdomen was not included as predictor in any of the models with delta AICc ≤2 and is therefore not included in the table. See Appendices S13‐S18 for full model summaries of the six models.

This study was carried out in accordance with ethical guidelines stated by the Norwegian Ministry of Agriculture and Food through the Animal Welfare Act, which does not require specific approvals for this type of investigation.

## 3 RESULTS

The mass of the carotenoid in the eggs of the 42 Arctic charr females quantified by HPLC was totally dominated by carotenoid with zeaxanthin spectrum, both free and esterified (Figure 1). The remaining carotenoid was minor amounts of degraded carotenoid with a wavelength <440 nm, and small amounts of non‐degraded carotenoid >440 nm with spectra like tunaxanthin, adonixanthin, idoxanthin, adonirubin, and astaxanthin (Figure 1, Appendix S8). After pooling all the different carotenoids, the mean (±SE) carotenoid content (including degraded carotenoid) in the eggs of our study population was 3.4 (±0.13, range 1.5–4.8) μg g^−1^ egg wet weight (*n* = 38) and mean (±SE) carotenoid content per egg was 160.4 (±6.32, range 90.7–251.2) ng egg^−1^ (*n* = 42).

**FIGURE 2:**
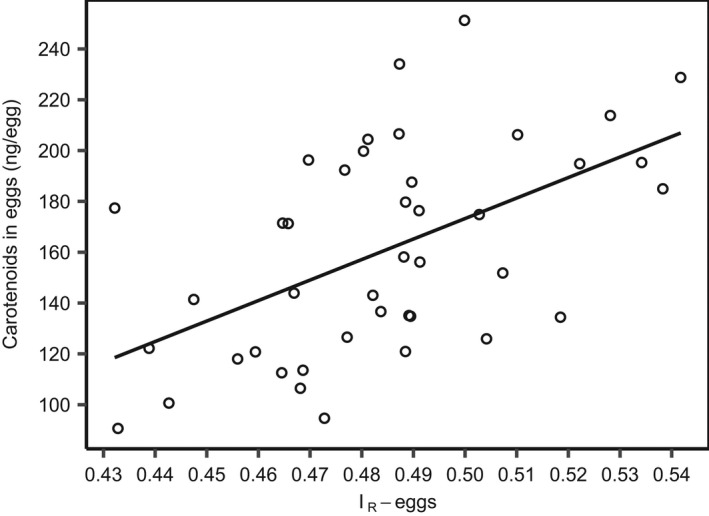
Scatter‐plot showing total mass of carotenoid in eggs (ng/egg) and degree of red (or yellowish) color in the eggs (*I*_R_ – eggs). The line represents the linear regression line (*y* = −230 + 807*x*)

**FIGURE 3:**
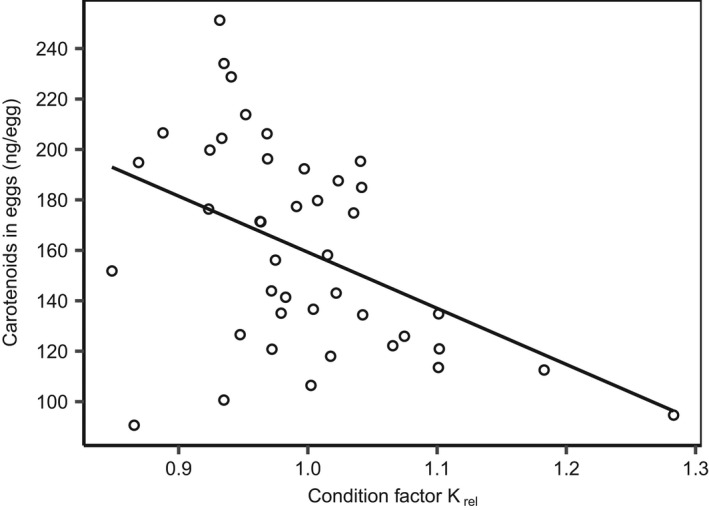
Scatter‐plot showing total mass of carotenoid in eggs (ng/egg) and the condition factor (*K*_rel_) of female Arctic charrs. The line represents the linear regression line (*y* = 382 − 222*x*)

As expected, eggs with a high mass of carotenoid (carotenoid‐by‐HPLC) had a stronger yellowish color (*I*_R_‐eggs) than eggs low in carotenoid (*R* ^2^ = .28, *p* < .0003, Figure 2). A significant negative relationship was found between the females' condition‐factor (*K_rel_*) and the total mass of carotenoid in the eggs (carotenoid‐by‐HPLC) (*R* ^2^ = .20, *p* = .003, Figure 3).

Intensity‐of‐red color at the females' abdomen (*I_R_*‐*abdomen*) was not associated with (i) carotenoid‐by‐HPLC in their eggs (*R* ^2^ = .004, *p* = .71, Appendix S9), (ii) intensity‐of‐red in their eggs (*I*_R_‐eggs) (*R* ^2^ = .002, *p* = .81, Appendix S10) nor with (iii) condition‐factor (*K*_rel_) (*R* ^2^ = .025, *p* = .32, Appendix S11). The association between the females' condition‐factor (*K*_rel_) and intensity of yellowish color of the eggs (intensity‐of‐red, *I_R_*‐eggs) was not significant (*R* ^2^ = .004, *p* = .70, Appendix S12).

**FIGURE 4:**
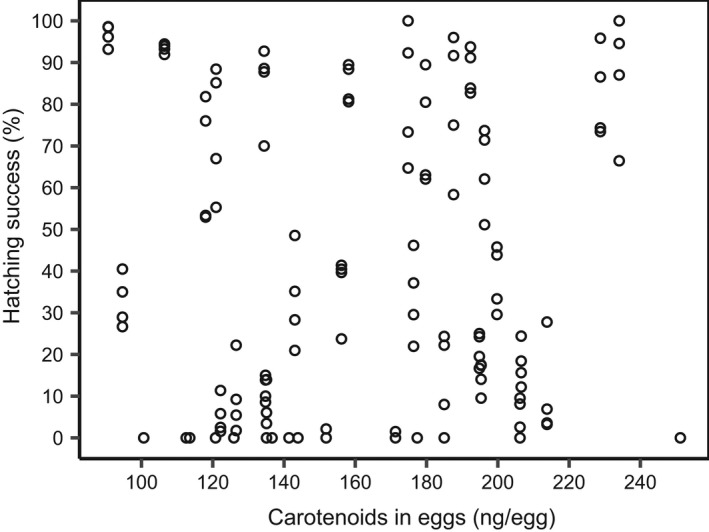
Scatter‐plot showing hatching success (%) and total mass of carotenoid in eggs (ng/egg)

**FIGURE 5:**
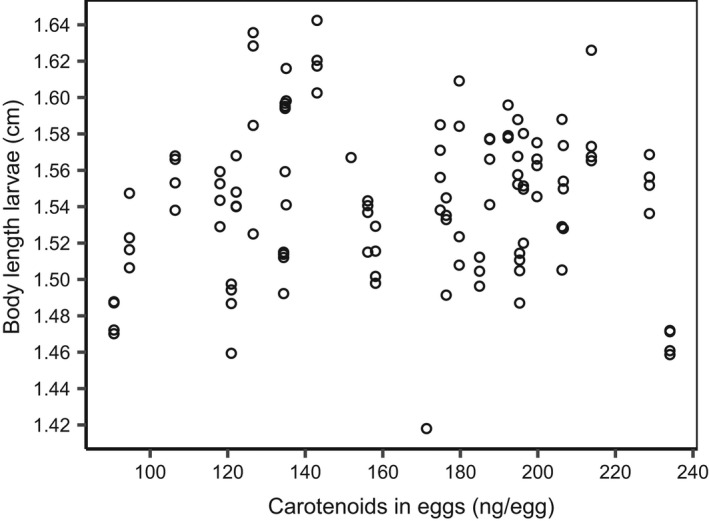
Scatter‐plot showing body length of larvae (cm) and total mass of carotenoid in eggs (ng/egg)

Tables 1 and 2 show test statistics from the combinations of predictors in the models to best explain the variation in each of the two response variables (i) hatching success of the eggs and (ii) length of larvae at hatching, respectively. The models included in each of the two tables are those with the lowest AICc and a delta AICc ≤2 (see Appendices S6 and S7 for models with delta >2). None of the models with different combinations of predictors of the eggs [carotenoid‐by‐HPLC, intensity‐of‐red eggs (*I*_R_‐eggs)], or the two other predictors of the female [intensity‐of‐red at the females' abdomen (*I*_R_‐abdomen)], or condition factor (*K_rel_*), had a significant explanatory effect on the variance in any of the two response variables (see Figures 4 and 5).

**TABLE 2:** Test statistics from linear mixed models with length of the newly hatched larvae as the response variable

Model	Intercept		df	logLik	AICc	Delta	Weight
	*β*	*p*					
1	1.54	<.0001	4	224.168	−440.0	0.00	0.469

Note

The table shows the one model with delta AICc ≤ 2. *β* represents the estimate and *p* is *p*‐value. None of the predictors (i) condition factor (*K*_rel_), (ii) the amount of carotenoid in eggs (carotenoid by HPLC), (iii) intensity‐of‐red abdomen (*I*_R_‐abdomen), and (iv) intensity‐of‐red eggs (*I*_R_‐eggs) were included in any of the models 3 with delta AICc ≤2, and are therefore not included in the table. See Appendix S19 for the full model summary.

## 4 DISCUSSION

None of the predictors (i) mass of carotenoid in the eggs, (ii) coloration of the eggs, (iii) color of the females' abdomen, or (iv) condition factor, had a significant explanatory effect on the variation of the two response variables “hatching success” or “length of larvae at hatching” in this experiment. Thus, our results demonstrated no effect of natural variation in carotenoid in unfertilized eggs on fitness parameters from fertilization to hatching in this population of wild Arctic charr.

Results from previous studies on this topic are mixed as outlined in the Introduction. For example, Wilkins et al. (2017) reported no effect on embryo mortality or hatching time by carotenoid content in unfertilized eggs of wild brown trout (*Salmo trutta*), which is similar to the result of the present study. Yet, after exposing the experimental groups of eggs to stress treatment, a positive effect of egg‐carotenoid on embryo survival was reported from the same study (Wilkins, da Cunha, et al., 2017) and two other studies on Salmonidae (Tyndale et al., 2008; Wilkins, Marques da Cunha, et al., 2017). Fifty‐five percent of the offspring survived until hatching in the present study. This is relatively high compared to the 34% survival estimate to the eye pigmentation stage of Arctic charr by Janhunen, Kekäläinen, et al. (2011), and the 40% survival of developing embryos from cultivated Arctic charr parents (Janhunen, Peuhkuri, et al., 2011). This suggests that the fertilized eggs and larvae in the present study have not been exposed to very high stress levels. Both concentration and composition of carotenoid in the egg have the potential to effect embryo development. The mean concentration of carotenoid in the Arctic charr eggs in the present study, 3.4 μg g^−1^ egg, is at a level suggested to improve offspring survival in salmonid fishes by Craik (1985) and Tyndale et al. (2008).

Astaxanthin, zeaxanthin, and lutein (720, 848 and 178 nM egg^−1^, respectively) dominated in the study by Wilkins, Marques da Cunha, et al. (2017), whereas the dominating carotenoid was astaxanthin in, for example, the study by Tyndale et al. (2008). As astaxanthin is considered 10 times as potent antioxidant compared to zeaxanthin (Ambati et al., 2014; Yuan et al., 2011), the dominance of the relatively low potent zeaxanthin and low levels of astaxanthin in the present study might have contributed to the lack of effect of the carotenoid on the fish larvae's survival. Thus, this suggests that the effect of egg‐carotenoid on offspring quality in the present study might have been different if the eggs were exposed to more stress, or if the natural carotenoid in the eggs contained a higher percentage of astaxanthins or other potent antioxidants, for example, by feeding the female parents *ad libitum* potent carotenoid before the experiment like in the studies Janhunen, Kekäläinen, et al. (2011). However, as this study examined naturally occurring egg carotenoid, feeding the parents with carotenoid would be in conflict with the aim of the study.

No association was revealed between the intensity of red color of the females' carotenoid‐based abdomen and mass of carotenoid in the eggs in the present study. This is in contrast to several previous studies reporting a negative association between the two parameters in three‐spined sticklebacks (*Gasterosteus aculeatus*) (Nordeide et al., 2006), sockeye salmon (*Oncorhynchus nerka*) (Ramstad et al., 2010), and brown trout (Wilkins, Marques da Cunha, et al., 2017) (see Introduction). This lack of negative association is also to some degree in contrast to negative association between intensity of carotenoid‐based ornamentation and offspring survivorship in female Arctic charr (Janhunen, Kekäläinen, et al., 2011). However, these two studies did not report any quantification of carotenoid or coloration of the charr eggs. Thus, it is uncertain if the eggs with poor survival from females with red carotenoid‐based ornamented abdomens, were also paler and lower in carotenoid, and vice versa. A previous study from the same Arctic charr population as in the present study reported a slightly nonsignificant negative association between conspicuousness of the carotenoid‐based female ornament intensity and their eggs (Nordeide et al., 2008). We can only speculate about reasons for this apparent inconsistency between the two studies from the same population. Examples of such speculations are slightly later sampling (between 2 and 9 days) in the present study compared to the first study (Nordeide et al., 2008), between‐years variation in onset of spawning, and gradual depletion of carotenoid in the eggs in later compared to earlier batches within a spawning season.

The Arctic charr eggs in this study had mainly carotenoid with a zeaxanthin‐like spectrum, in opposition to the egg carotenoid reported by Nordeide et al. (2008), which was reported as mainly carotenoid with lutein‐like spectrum. Minor amounts of other carotenoids were present in both studies. Due to this difference in observations of the same species caught from the same lake, the raw HPLC data from both experiments were reexamined. This reexamination revealed zeaxanthin as the major carotenoid in the eggs from both studies, compared to analyses of commercial standards. Thus, zeaxanthin was the main carotenoid also in the Nordeide et al. (2008) study.

The ovaries of five species of *Salvelinus* sp. (*S*. *alpinus* not included) contained idoxanthin as the major carotenoid, together with β,β‐carotene tetrol, zeaxanthin, lutein, and a few more (Ando et al., 1989). The specimens in this experiment were fed feed supplemented by Antarctic krill. In another experimental study Arctic charr were fed astaxanthin‐supplemented feed (Bjerkeng et al., 2000). More than half of the eggs' carotenoid consisted of idoxanthin, together with crustaxanthin (β,β‐carotene‐3,4,3′,4′‐tetrol), and unidentified carotenoid (Bjerkeng et al., 2000). Crustaxanthin has a similar absorption spectrum as zeaxanthin, but with significantly higher polarity than zeaxanthin. The difference in carotenoid of the eggs/ovaries between the present study with wild specimens and those fed by artificial feed (Ando et al., 1989; Bjerkeng et al., 2000) may be due to differences in their feed and differences between *Salvelinus* species and populations.

The empirical support for the evolution of female ornaments by mate choice is ambiguous and the evolution of ornaments in females remains challenging to explain. If ornaments signal genetic quality of the females (e.g., Zahavi, 1975) or direct advantages of non‐genetic maternal resources (Blount et al., 2000), male mate choice for ornamented females might be adaptive. On the other hand, females of some species allocating resources to ornaments may do so at the expense of resources available for offspring and thus constrain their fitness (Fitzpatrick et al., 1995). In species with a negative relationship between ornament and egg quality or fecundity, one may argue that the benefit of the ornament may not outweigh its fitness cost. Males choosing to fertilize eggs from such ornamented females may sire offspring of relatively poor quality (Fitzpatrick et al., 1995). This is complicated by the argument that even in species where female ornaments are negatively associated with offspring fitness, intensely ornamented high‐quality females may potentially still have higher fitness than drab females (Simmons & Emlen, 2008; Zahavi, 1975). For this to occur, (i) higher sexual attractiveness of the ornamented mother's offspring must compensate her reduced number of surviving offspring, (ii) or if high quality females suffer less reduction in offspring number or quality from the ornament than females of low quality. If so, it may still be adaptive for males to choose the most ornamented females as mates (Simmons & Emlen, 2008; Watson & Simmons, 2010; Zahavi, 1975). The outcome of the conflicting scenarios under a negative association between ornament and offspring, will depend on whether the attractiveness of the ornament outweighs the fecundity cost of the ornament (Fitzpatrick et al., 1995; LeBas, 2006; Watson & Simmons, 2010). Future selection experiments over several generations might reveal whether it is adaptive for males to choose ornamented or drab females (Nordeide et al., 2013). If it turns out that this varies between species, between populations or points in time, we potentially have an explanation for the conflicting results as revealed by the reviews as referred to above.

To conclude, this study does not demonstrate any negative or positive effect of the intensity of the carotenoid‐based ornament of female Arctic charr on the quality of their eggs and larvae. No association is not as expected from predictions of the “direct selection hypothesis” (see Introduction). This result is not in conflict with the “genetic correlation hypothesis,” although lack of significant association between parameters is obviously not a bold prediction.

## CONFLICT OF INTEREST

None declared.

## AUTHOR CONTRIBUTIONS

**Torvald Blikra Egeland:** Conceptualization (equal); Data curation (lead); Formal analysis (lead); Funding acquisition (supporting); Investigation (lead); Methodology (equal); Project administration (equal); Resources (equal); Software (lead); Supervision (equal); Validation (equal); Visualization (equal); Writing – original draft (equal); Writing – review & editing (equal). **Einar Skarstad Egeland:** Conceptualization (supporting); Data curation (equal); Formal analysis (equal); Funding acquisition (supporting); Investigation (equal); Methodology (equal); Project administration (supporting); Resources (equal); Software (supporting); Supervision (equal); Validation (equal); Visualization (equal); Writing – original draft (equal); Writing – review & editing (equal). **Jarle Tryti Nordeide:** Conceptualization (lead); Data curation (supporting); Formal analysis (equal); Funding acquisition (lead); Investigation (equal); Methodology (equal); Project administration (equal); Resources (lead); Software (supporting); Supervision (equal); Validation (equal); Visualization (equal); Writing – original draft (lead); Writing – review & editing (equal).

## Supporting information

Supplementary Material

## Data Availability

The data presented in this study are archived at Dryad https://datadryad.org/stash/share/zl_MczFntpxP_ie9OfOSGtRd8L15XPzjAByifa7SWJ4; https://doi.org/10.5061/dryad.rbnzs7hcw.
