# Supplementary material for: Does egg carotenoid improve larval quality in Arctic charr (Salvelinus alpinus)?
Source: Ecol Evol. 2022 Apr 11;12(4):e8812. doi: 10.1002/ece3.8812 (PMC9001117; doi:10.1002/ece3.8812)
Supplement: Supplementary file 1 — Supplementary Material [file ECE3-12-e8812-s001.docx]

Appendix


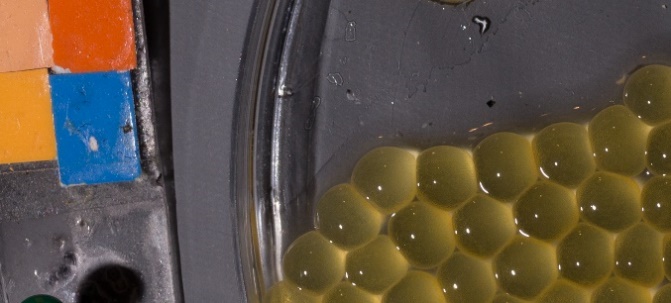

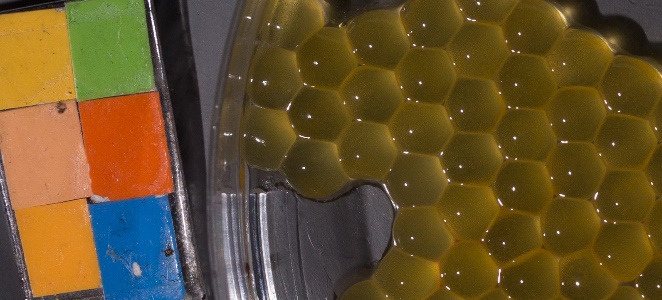


Appendix S1. Pictures showing the colour difference between pale eggs (left) and red eggs (right). The eggs on the left picture had I_R_ = 0.43 and a total carotenoids mass of 177.4 ng/egg. The eggs on the right picture had I_R_ = 0.49 and a total carotenoids mass of 234.1 ng/egg (see also App. 3 and 4).


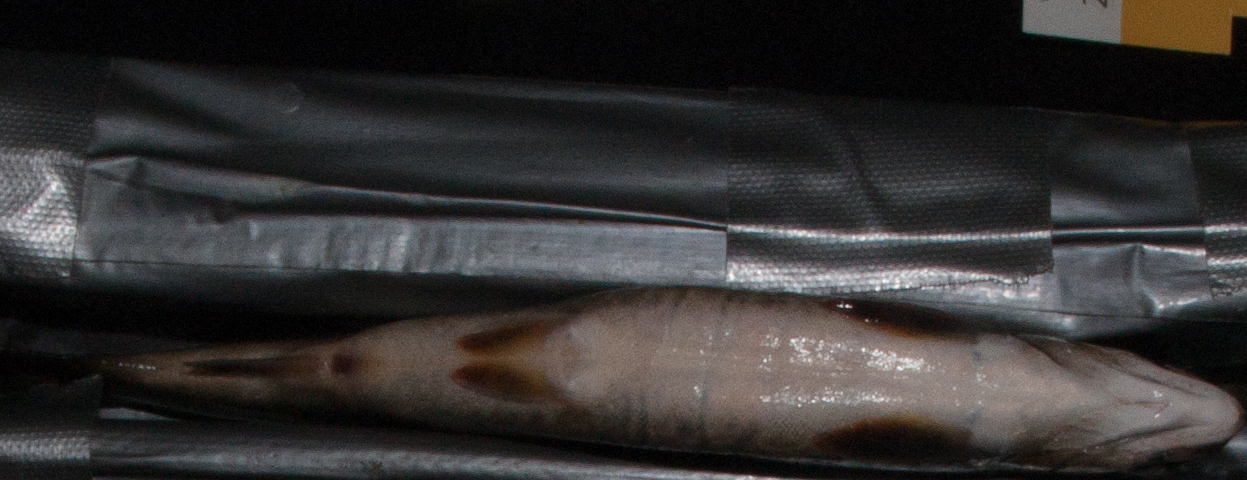

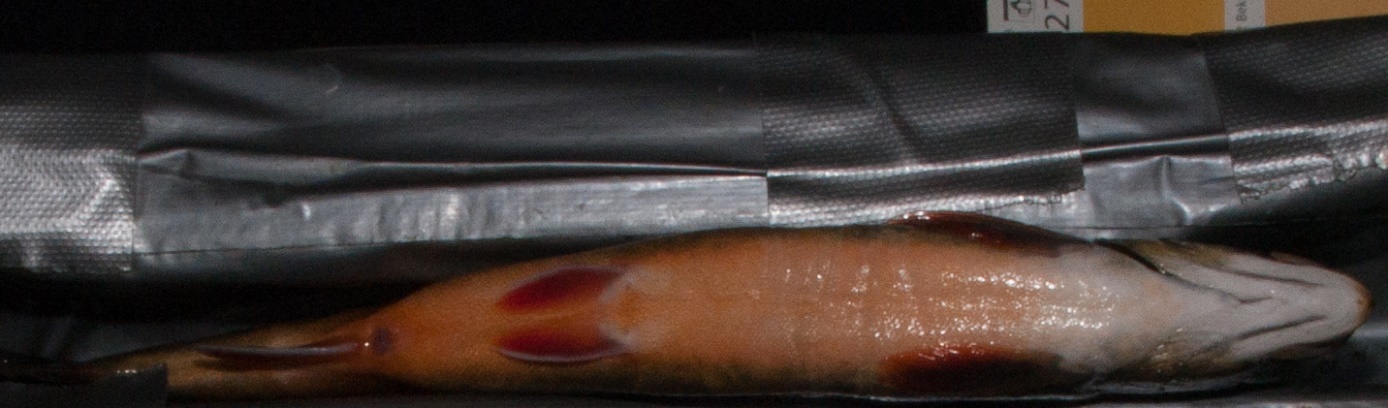


Appendix S2. Pictures illustrating the contrast in colouration of the abdomen of the females. The red intensity of the colour of the abdomen for the two females was I_R_ = 0.37 (top) and I_R_ = 0.54 (bottom).


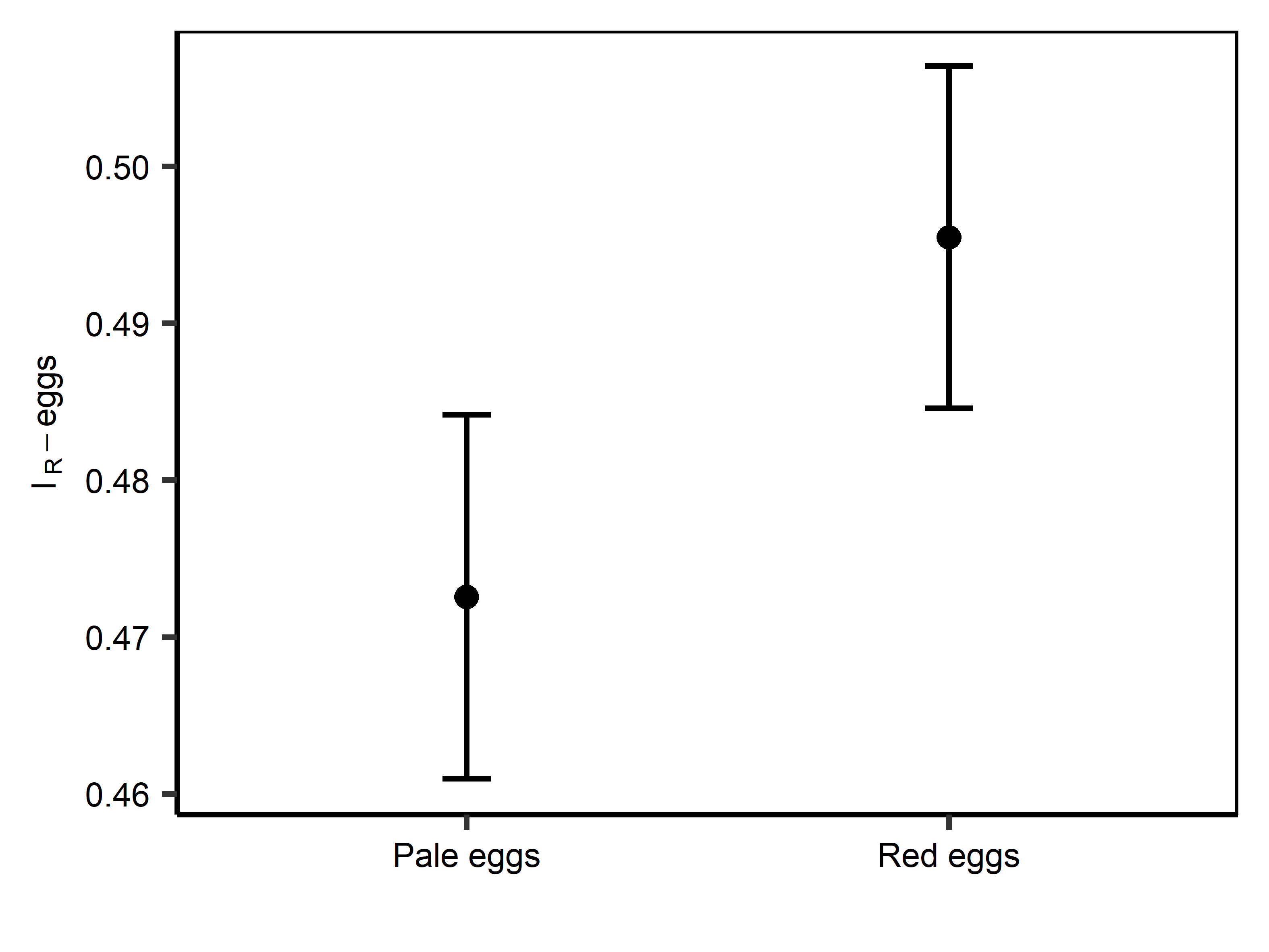


Appendix S3. Intensity of red (I_R_-eggs, mean ± 95% CI) of pale and red eggs. The eggs from two females in a pair of females were first categorized as either “Pale” or “Red” by the eyes (“colour by vision”). Then the mean I_R_-value was calculated from the category of females with “Pale” eggs and “Red” eggs separately.


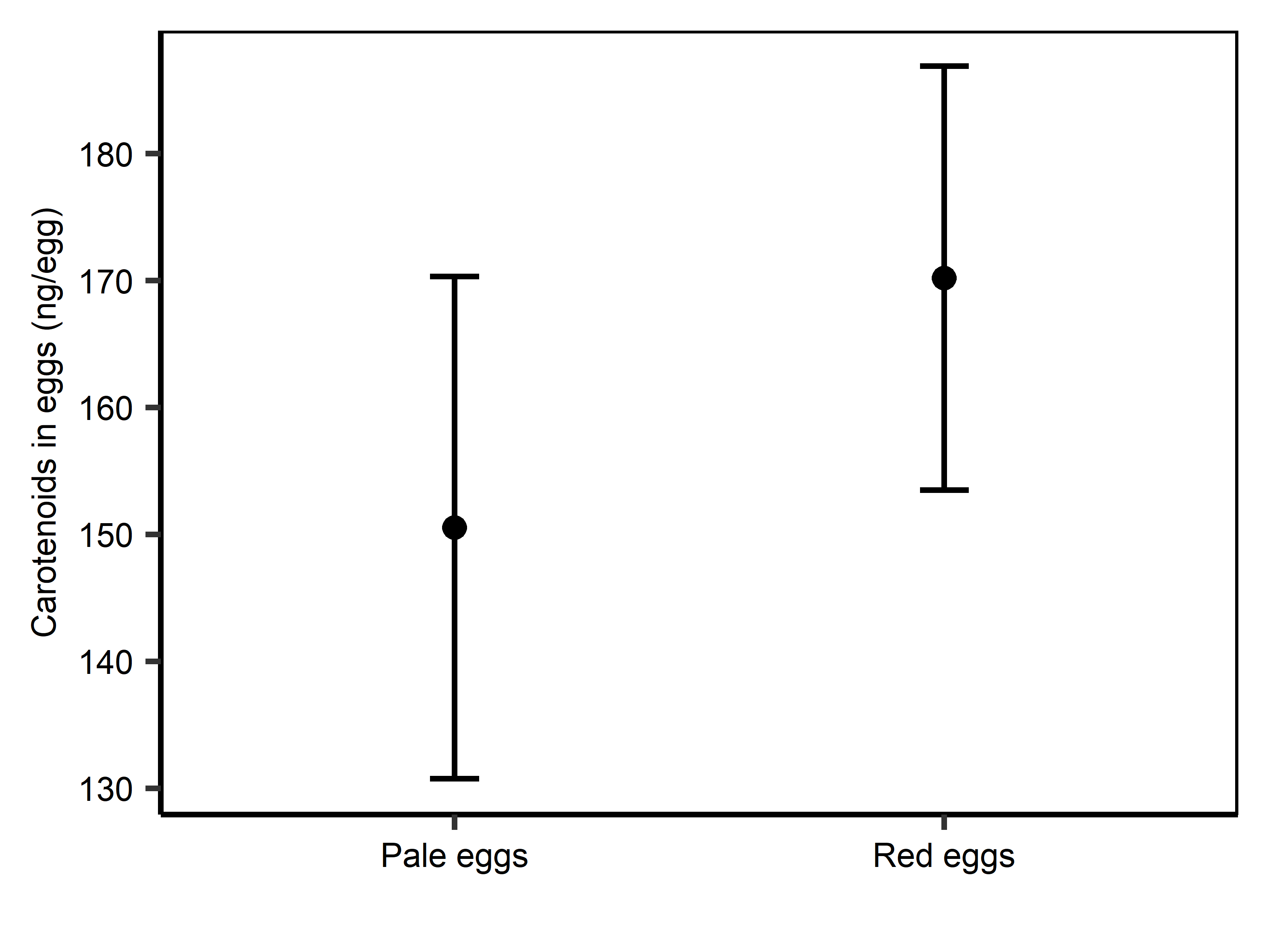


Appendix S4. Mass of carotenoids per egg (mean ± 95 % CI) in pale and red eggs. The eggs from two females in a pair of females were first categorized as either “Pale” or “Red” (“colour by vision”). Then the mean value from the HPLC-analyses was calculated from the category of females with “Pale” eggs and “Red” eggs separately.

Appendix S5. Hatchery diary showing the number of eggs/eyelings/larvaes that were removed (because of mortality and/or because of unfertilized eggs) weekly. A total of 2931 larvae were used to measure larvae length and hatching success.

|  | **1st week** | **2nd week** | **3rd week** | **4th week** | **5th week** | **6th week** | **7th week** | **8th week** | **9th week** | **10th week** | **Sum** |
| --- | --- | --- | --- | --- | --- | --- | --- | --- | --- | --- | --- |
| **Eggs removed** | 0 | 939 | 292 | 607 | 821 | 596 | 438 | 530 | 1103 | 12 | 5338 |
| **Eyelings removed** |  |  |  |  |  |  |  | 7 |  | 4 | 11 |
| **Larvae removed** |  |  |  |  |  |  |  | 3 | 1 | 16 | 20 |
| **Number of larvae** |  |  |  |  |  |  |  |  |  |  | 2931 |

| Model | K_rel_ | I_R_-eggs | Carotenoids-by-HPLC | K_rel_ x I_R_-eggs | K_rel_ x Carotenoids-by-HPLC | I_R_-eggs x Carotenoids-by-HPLC | K_rel_ x  I_R_-eggs x Carotenoids-by-HPLC | df | logLik | AICc | delta | weight |
| --- | --- | --- | --- | --- | --- | --- | --- | --- | --- | --- | --- | --- |
| 7 | X |  | X |  |  |  |  | 5 | -453.013 | 916.4 | 2.52 | 0.053 |
| 8 | X | X |  |  | X |  |  | 6 | -452.038 | 916.6 | 2.73 | 0.048 |
| 9 | X |  | X | X |  |  |  | 6 | -452.259 | 917.1 | 3.17 | 0.038 |
| 10 | X | X | X |  |  |  |  | 6 | -452.633 | 917.8 | 3.91 | 0.026 |
| 11 |  | X | X |  |  | X |  | 6 | -452.714 | 918.0 | 4.08 | 0.024 |
| 12 | X | X | X | X |  |  |  | 7 | -451.821 | 918.4 | 4.48 | 0.020 |
| 13 | X | X | X |  | X |  |  | 7 | -451.863 | 918.5 | 4.56 | 0.019 |
| 14 | X | X | X |  |  | X |  | 7 | -452.624 | 920.0 | 6.08 | 0.009 |
| 15 | X | X | X | X | X |  |  | 8 | -451.707 | 920.4 | 6.47 | 0.007 |
| 16 | X | X | X | X |  | X |  | 8 | -451.782 | 920.5 | 6.62 | 0.007 |
| 17 | X | X | X |  | X | X |  | 8 | -451.826 | 920.6 | 6.70 | 0.007 |
| 18 | X | X | X | X | X | X |  | 9 | -451.662 | 922.5 | 8.62 | 0.003 |
| 19 | X | X | X | X | X | X | X | 10 | -451.366 | 924.2 | 10.31 | 0.001 |

Appendix S6. List of models with egg hatching success as the response variable. The list contains models with delta Akaike’s information criteria (AICc) > 2 (see Table 1 for models with AICc ≤ 2). The x’s indicates witch of the predictors, (i) condition factor (K_rel_), (ii) intensity-of-redd eggs (I_r_-eggs), and (iii) the amount of carotenoids in eggs (carotenoids-by-HPLC), the different models includes

Appendix S7. List of models with egg hatching success as the response variable. The list contains models with delta Akaike’s information criteria (AICc) > 2 (see Table 1 for models with AICc ≤ 2). The x’s indicates witch of the predictors, (i) condition factor (K_rel_), (ii) intensity-of-redd eggs (I_r_-eggs), and (iii) the amount of carotenoids in eggs (carotenoids-by-HPLC), the different models includes.

| Model | K_rel_ | I_R_-eggs | Carotenoids-by-HPLC | K_rel_ x I_R_-eggs | K_rel_ x Carotenoids-by-HPLC | I_R_-eggs x Carotenoids-by-HPLC | K_rel_ x  I_R_-eggs x Carotenoids-by-HPLC | df | logLik | AICc | delta | weight |
| --- | --- | --- | --- | --- | --- | --- | --- | --- | --- | --- | --- | --- |
| 2 | X | X |  | X |  |  |  | 7 | 226.187 | -440.0 | 2.67 | 0.161 |
| 3 |  | X |  |  |  |  |  | 5 | 223.859 | -437.1 | 2.81 | 0.151 |
| 4 | X |  |  |  |  |  |  | 5 | 222.906 | -435.2 | 4.72 | 0.058 |
| 5 | X | X |  |  |  |  |  | 6 | 222.689 | -432-6 | 7.39 | 0.015 |
| 6 |  |  | X |  |  |  |  | 5 | 216.494 | -422.4 | 17.54 | 0.000 |
| 7 |  | X | X |  |  |  |  | 6 | 216.583 | -420.4 | 19.6 | 0.000 |
| 8 | X | X | X | X |  |  |  | 7 | 218.853 | -420.3 | 19.66 | 0.000 |
| 9 | X |  | X |  |  |  |  | 6 | 215.274 | -417.7 | 22.22 | 0.000 |
| 10 | X | X | X |  |  |  |  | 7 | 215.379 | -415.7 | 24.29 | 0.000 |
| 11 | X | X | X | X | X |  |  | 9 | 215.542 | -411.4 | 28.6 | 0.000 |
| 12 |  | X | X |  |  | X |  | 7 | 212.549 | -410.0 | 29.95 | 0.000 |
| 13 | X | X | X | X |  | X |  | 9 | 214.866 | -410.0 | 30.01 | 0.000 |
| 14 | X | X | X |  |  | X |  | 8 | 211.311 | -405.2 | 34.75 | 0.000 |
| 15 | X |  | X |  | X |  |  | 7 | 210.027 | -405.0 | 34.99 | 0.000 |
| 16 | X | X | X |  | X |  |  | 8 | 210.071 | -402.7 | 37.23 | 0.000 |
| 17 | X | X | X | X | X | X |  | 10 | 211.537 | -400.9 | 39.09 | 0.000 |
| 18 | X | X | X | X | X | X | X | 11 | 210.458 | -396.3 | 43.71 | 0.000 |
| 19 | X | X | X |  | X | X |  | 9 | 206.023 | -396.3 | 47.70 | 0.000 |

Appendix S8. Table showing the total mass (ng/egg) of the different carotenoids in the eggs (all eggs are pooled) and the proportion of each carotenoids (%) in relation to the total mass.

|  | **Degraded carotenoids** | | | | | | | | | | **Carotenoids** | | | | | |
| --- | --- | --- | --- | --- | --- | --- | --- | --- | --- | --- | --- | --- | --- | --- | --- | --- |
| **Wavelength (nm)** | **<350** | **350** | **360** | **370** | **380** | **390** | **400** | **410** | **420** | **430** | **408** | **440** | **446** | **450** | **465** | **475** |
| **Total mass (ng/egg)** | 2.99 | 2.55 | 16.91 | 36.41 | 232.08 | 56.87 | 480.02 | 253.02 | 366.52 | 3.51 | 12.06 | 14.53 | 62.31 | 4698.29 | 455.22 | 42.95 |
| **Proportion of content (%)** | 0.04 | 0.04 | 0.25 | 0.54 | 3.45 | 0.84 | 7.13 | 3.76 | 5.44 | 0.05 | 0.18 | 0.22 | 0.92 | 69.75 | 6.76 | 0.64 |


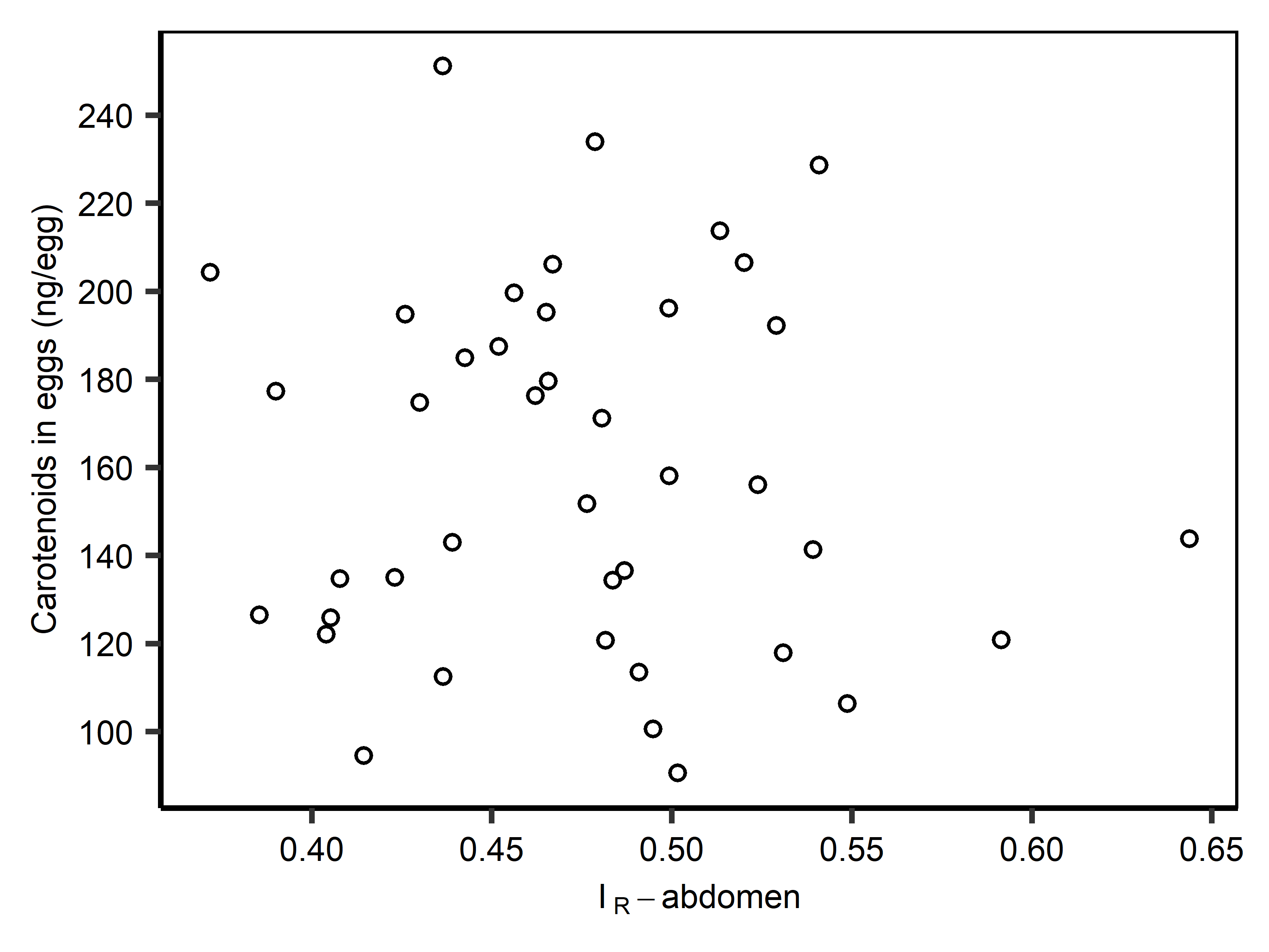


Appendix S9. Scatter-plot between total mass of carotenoids (ng/egg) and red intensity of the abdomen (I_r_-abdomen) in Arctic charr females.


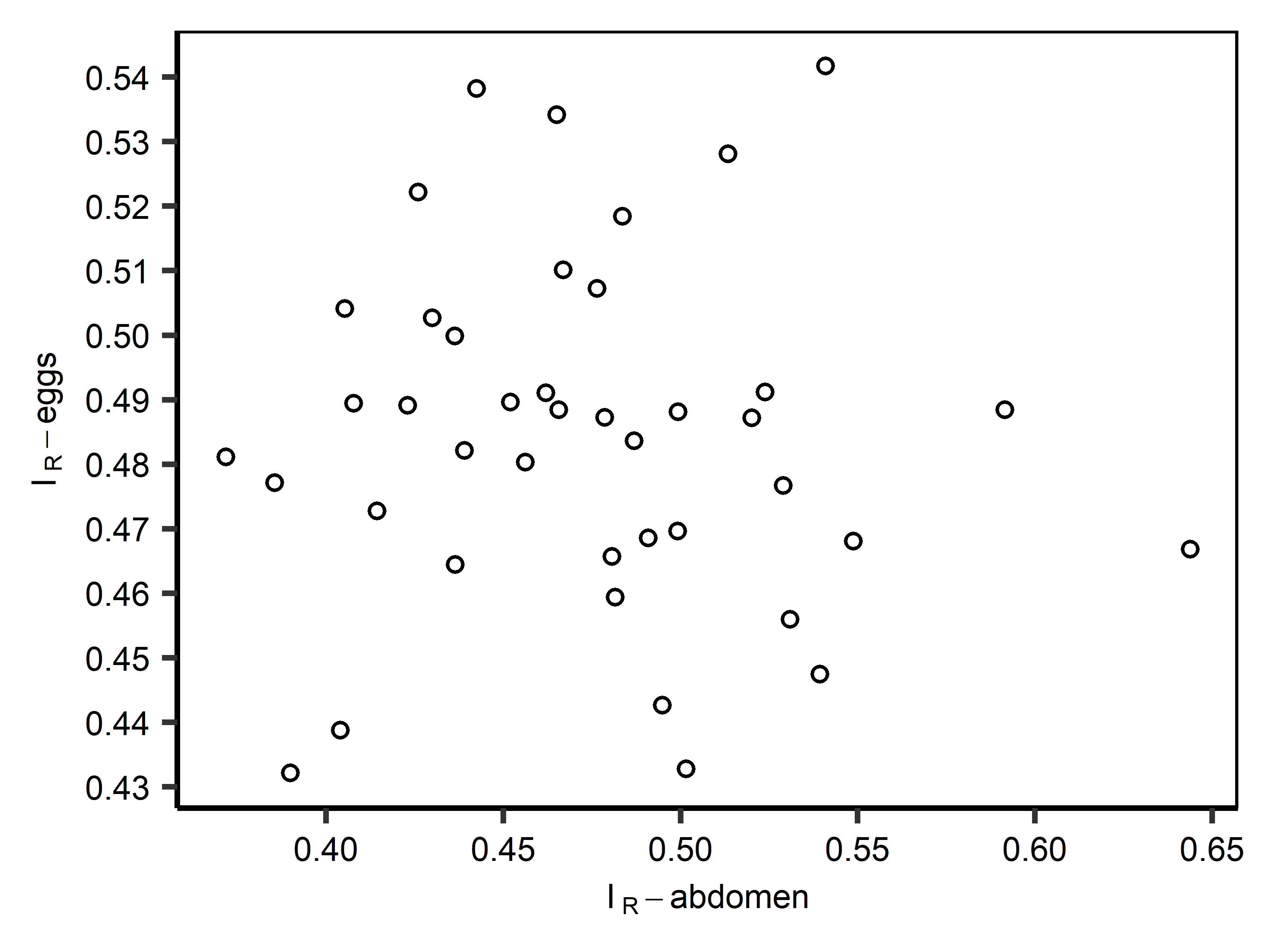


Appendix S10. Scatter-plot with the intensity of red at the abdomen (intensity-of-red abdomen, *I_R_*-abdomen) plotted against intensity of red-yellowish colour of the eggs (intensity-of-eggs, *I_R_*-eggs).


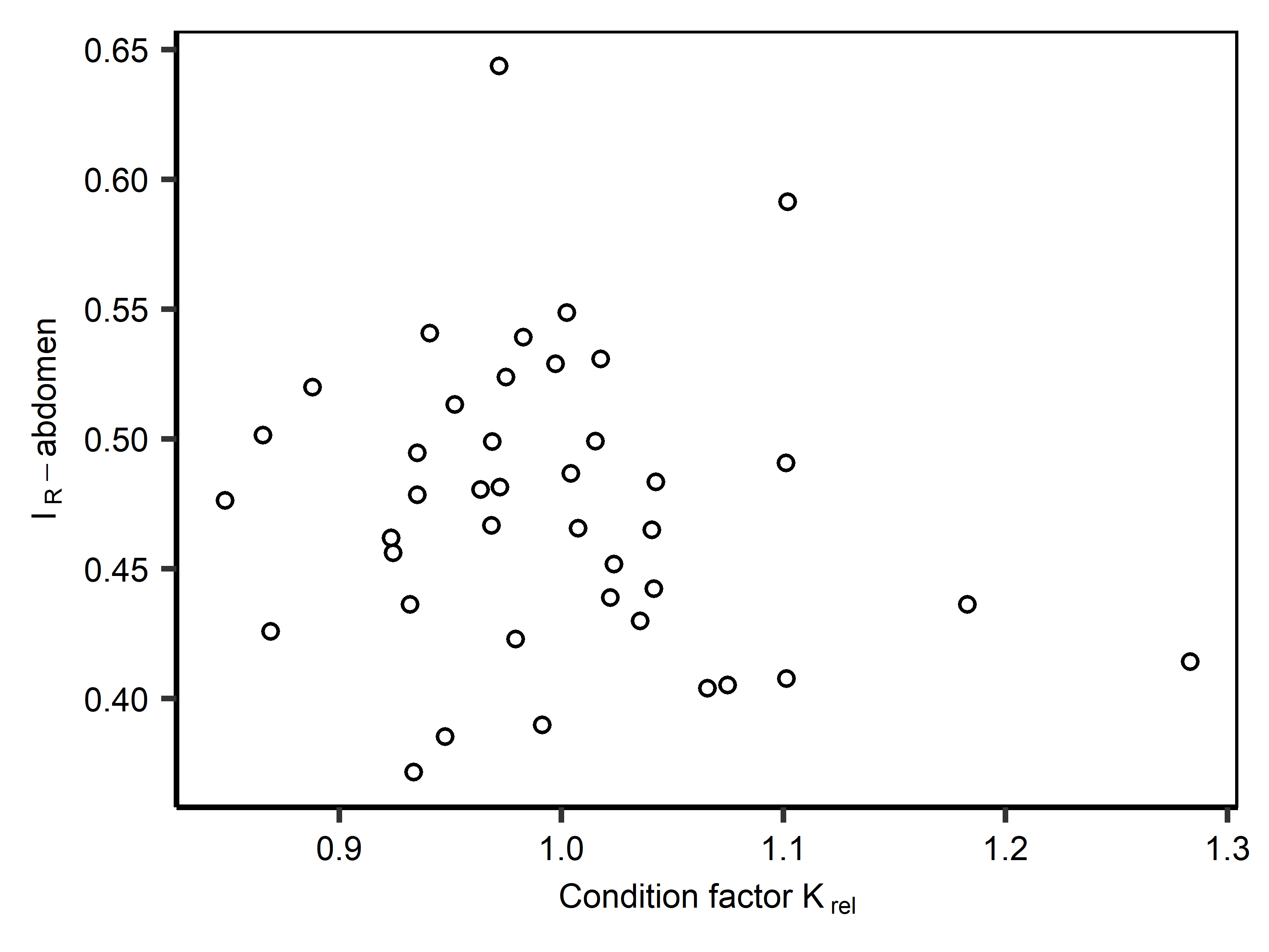


Appendix S11. Scatter-plot with the intensity of red at the abdomen (*I_R_*-abdomen) plotted against condition factor (K_rel_).


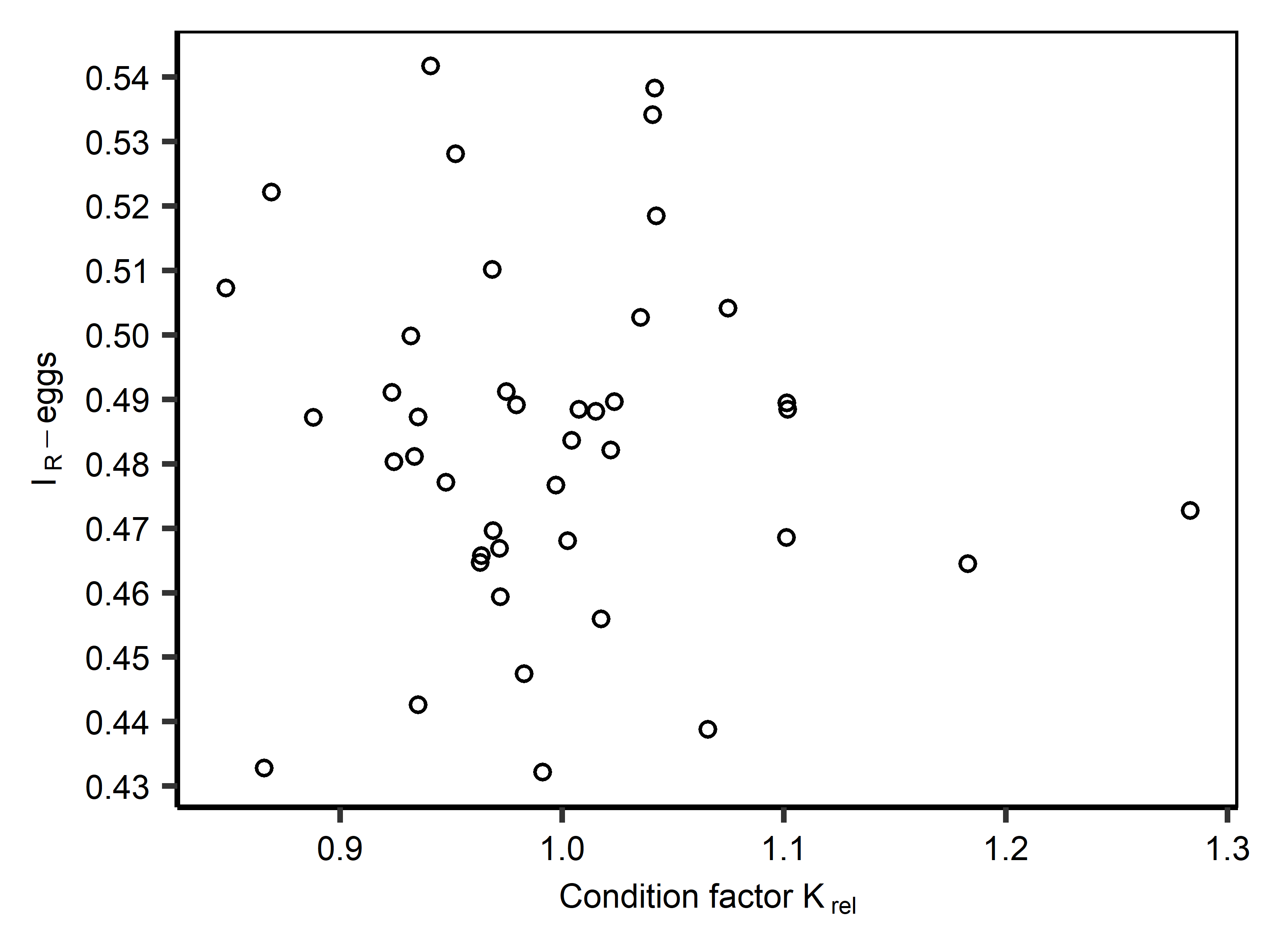


Appendix S12. Scatter-plot with the condition factor (K_rel_) plotted against intensity of red-yellowish colour of the eggs (*I_R_*-eggs).

Appendix S13. Model summary of model 1 (table 1). Fixed effects are presented with estimates parameters including standard error (St. error), 95 % confidence intervals (95 % CI) and *p*-values (*p*).

| **Response** | **Predictor** | **Estimate** | **St. error** | **95 % CI** | ***p*** |
| --- | --- | --- | --- | --- | --- |
| Hatchery success | Intercept | -2.15 | 0.68 | -3.48 to -0.81 | 0.0017 |

Appendix S14. Model summary of model 2 (table 1). Fixed effects are presented with estimates parameters including standard error (St. error), 95 % confidence intervals (95 % CI) and *p*-values (*p*).

| **Response** | **Predictor** | **Estimate** | **St. error** | **95 % CI** | ***p*** |
| --- | --- | --- | --- | --- | --- |
| Hatchery success | Intercept | -16.53 | 10.35 | -36.81 to 3.75 | 0.110 |
|  | I_r_-eggs | 29.63 | 21.27 | -12.06 to 71.32 | 0.164 |

Appendix S15. Model summary of model 3 (table 1). Fixed effects are presented with estimates parameters including standard error (St. error), 95 % confidence intervals (95 % CI) and *p*-values (*p*).

| **Response** | **Predictor** | **Estimate** | **St. error** | **95 % CI** | ***p*** |
| --- | --- | --- | --- | --- | --- |
| Hatchery success | Intercept | -4.68 | 2.08 | -8.76 to -0.61 | 0.0244 |
|  | Carotenoids by HPLC | 0.02 | 0.01 | -0.01 to 0.04 | 0.1945 |

Appendix S16. Model summary of model 4 (table 1). Fixed effects are presented with estimates parameters including standard error (St. error), 95 % confidence intervals (95 % CI) and *p*-values (*p*).

| **Response** | **Predictor** | **Estimate** | **St. error** | **95 % CI** | ***p*** |
| --- | --- | --- | --- | --- | --- |
| Hatchery success | Intercept | 3.44 | 6.33 | -8.98 to 15.85 | 0.587 |
|  | K_rel_ | -5.60 | 6.32 | -17.98 to 6.79 | 0.376 |

Appendix S17. Model summary of model 5 (table 1). Fixed effects are presented with estimates parameters including standard error (St. error), 95 % confidence intervals (95 % CI) and *p*-values (*p*).

| **Response** | **Predictor** | **Estimate** | **St. error** | **95 % CI** | ***p*** |
| --- | --- | --- | --- | --- | --- |
| Hatchery success | Intercept | -10.95 | 12.93 | -36.29 to 14.38 | 0.397 |
|  | K_rel_ | -4.60 | 6.39 | -17.13 to 7.93 | 0.472 |
|  | I_r_-eggs | 27.60 | 21.68 | -14.89 to 70.10 | 0.203 |

Appendix S18. Model summary of model 6 (table 1). Fixed effects are presented with estimates parameters including standard error (St. error), 95 % confidence intervals (95 % CI) and *p*-values (*p*).

| **Response** | **Predictor** | **Estimate** | **St. error** | **95 % CI** | ***p*** |
| --- | --- | --- | --- | --- | --- |
| Hatchery success | Intercept | -13.62 | 11.12 | -35.41 to 8.17 | 0.220 |
|  | I_r_-eggs | 20.52 | 25.06 | -28.60 to 69.65 | 0.413 |
|  | Carotenoids by HPLC | 0.01 | 0.01 | -0.02 to 0.04 | 0.516 |

Appendix S19. Model summary of model 1 (table 2). Fixed effects are presented with estimates parameters including standard error (St. error), 95 % confidence intervals (95 % CI) and *p*-values (*p*).

| **Response** | **Predictor** | **Estimate** | **St. error** | **95 % CI** | ***p*** |
| --- | --- | --- | --- | --- | --- |
| Length larvae | Intercept | 1.54 | 0.01 | 1.52 to 1.55 | <0.0001 |
